# Narrative-based computational modelling of the Gp130/JAK/STAT signalling pathway

**DOI:** 10.1186/1752-0509-3-40

**Published:** 2009-04-15

**Authors:** Maria Luisa Guerriero, Anna Dudka, Nicholas Underhill-Day, John K Heath, Corrado Priami

**Affiliations:** 1 Laboratory for Foundations of Computer Science, School of Informatics, University of Edinburgh, Informatics Forum, 10 Crichton Street, EH8 9AB, Edinburgh, UK; 2 CRUK Growth Factor Group, School of Biosciences, University of Birmingham, Edgbaston, B15 2TT, UK; 3 Dip. di Ingegneria e Scienza dell'Informazione, Università di Trento, Via Sommarive 14, 38100, Povo (Trento), Italy; 4 The Microsoft Research – University of Trento Centre for Computational and Systems Biology, Piazza Manci 17, 38100, Povo (Trento), Italy

## Abstract

**Background:**

Appropriately formulated quantitative computational models can support researchers in understanding the dynamic behaviour of biological pathways and support hypothesis formulation and selection by "in silico" experimentation. An obstacle to widespread adoption of this approach is the requirement to formulate a biological pathway as machine executable computer code. We have recently proposed a novel, biologically intuitive, narrative-style modelling language for biologists to formulate the pathway which is then automatically translated into an executable format and is, thus, usable for analysis via existing simulation techniques.

**Results:**

Here we use a high-level narrative language in designing a computational model of the gp130/JAK/STAT signalling pathway and show that the model reproduces the dynamic behaviour of the pathway derived by biological observation. We then "experiment" on the model by simulation and sensitivity analysis to define those parameters which dominate the dynamic behaviour of the pathway. The model predicts that nuclear compartmentalisation and phosphorylation status of STAT are key determinants of the pathway and that alternative mechanisms of signal attenuation exert their influence on different timescales.

**Conclusion:**

The described narrative model of the gp130/JAK/STAT pathway represents an interesting case study showing how, by using this approach, researchers can model biological systems without explicitly dealing with formal notations and mathematical expressions (typically used for biochemical modelling), nevertheless being able to obtain simulation and analysis results. We present the model and the sensitivity analysis results we have obtained, that allow us to identify the parameters which are most sensitive to perturbations. The results, which are shown to be in agreement with existing mathematical models of the gp130/JAK/STAT pathway, serve us as a form of validation of the model and of the approach itself.

## Background

Biological signalling pathways of even modest complexity cannot be comprehensively analysed within a feasible timescale by currently available experimental tools. However appropriate pathway models can be used to generate, explore and refine hypotheses guiding the formulation and prioritisation of experimental interventions. This has conventionally been approached by the use of models inspired by chemical kinetics and articulated mathematically in the form of ordinary differential equations. Recently an alternative approach, "molecules as computation", has been proposed in which a pathway is formulated as an executable computer programme [1,2] which can be interrogated to determine the dynamic behaviour, robustness and parameter sensitivities of the model [3]. The outcomes of in silico experimentation on the computer model can then be used to inform the design of biological interventions in vitro. Among the various computational languages which have been used to model biochemical systems, we consider here process calculi (see for example [4-8], or [9] for a review of the process calculi approach). These calculi are formal languages which allow modellers to perform several kinds of analyses on the models (e.g. model-checking, causality and equivalence analysis).

One key challenge of this approach is the accurate description of biological pathways in the form of an executable computer language. From the biologist's perspective the formulation has to capture the biologically interesting features of the pathway and be readily understood by other biologists. From the computer scientist's perspective the formulation has to conform to the rules of formal methods in computer science: it must be logically precise and unambiguous. There is therefore a potential language gap between what the biologist understands and what the computer model encodes. We have recently described a high-level biologically-intuitive textual language in which the signalling pathway is articulated in the form of a narrative of events concerning the interactions between components located in different compartments [10]. This articulation of the pathway is then translated into an executable computer programme (written in the process calculus-based BlenX language [11]) for further analysis. In this paper we describe, develop and interrogate such an executable model of the gp130/JAK/STAT signalling pathway [12] using the Narrative Language approach and explore its predictions by in silico experimentation.

**Figure 1:**
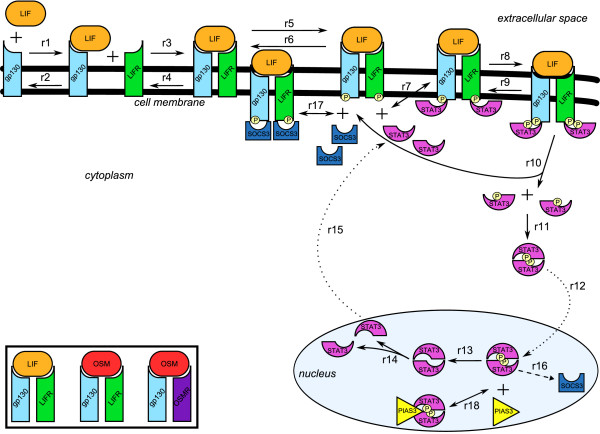
**Graphical representation of the gp130/JAK/STAT pathway**. Solid arrows represent biochemical reactions, dotted arrows represent transports, and dashed arrows represent syntheses. In the insert the different types of receptor complexes are shown.

The gp130/JAK/STAT signalling pathway (see for example [12]) is the subject of significant clinical and biological interest, not least due to the key role it plays in human fertility, neuronal repair, haematological development and embryonic stem cell renewal [13]. Members of the gp130 cytokine family, such as LIF or OSM, bind to the common signal transducing receptor chain gp130 and a second signalling receptor LIFR or OSMR [14]. Homo- or hetero-dimerisation of gp130, LIFR and OSMR induces activation of the receptor associated kinase JAK which in turn phosphorylates the latent transcription factor STAT which, as a consequence, undergoes homo-dimerisation, translocates to the nucleus and activates the transcription of downstream gene targets (Figure 1). Several features of this pathway make it an attractive case study for a computer programme based modelling approach. A characteristic feature of the gp130/JAK/STAT system is the role of spatial confinement in which the transcription factor STAT undergoes nuclear/cytoplasmic shuttling which is regulated by JAK-mediated phosphorylation at the plasma membrane and T-Cell Protein Tyrosine Phosphatase (TC-PTP)-mediated de-phosphorylation in the nucleus [15,16]. Aside from gp130/JAK activation by ligand the dynamics of the pathway can be regulated by a variety of mechanisms, which include STAT-mediated induction of Suppressor of Cytokine Signalling (SOCS) family proteins, which suppress JAK activation [17] and the Protein Inhibitor of Activated STAT (PIAS), an E3 family ubiquitin ligase which induces proteolytic degradation of phospho-STAT [18,19]. Chronic nuclear STAT activation and/or JAK activation [20,21] have been implicated as a predisposing event in a variety of tumour types indicating that pathway dynamics have significant impact on cell behaviour. Elucidating the relative influence of different pathway parameters on the activity of STAT will guide the evaluation of therapeutic interventions.

Much experimental data is available on the gp130/JAK/STAT pathway and several mathematical models have been developed based on ordinary differential equations (see for example [22-24]). We present a different approach to modelling this pathway based on formal computational methods. We use a novel textual language for modelling biochemical systems [10], and perform simulations through the BetaWB simulator [25], an existing tool based on Gillespie's stochastic simulation algorithm [26]. A key feature of the formal computational approach is the ability to rigorously explore, by in silico experimentation, the dynamic behaviour of the model to determine both the role of signal modifiers such as SOCS and the importance of parameter values. We validate our computational model by showing that it produces outputs which conform to those produced by experiment. We then perform in silico experiments on the model to determine first and second order parameter sensitivities and the effects of various types of pathway modulators. We show that the dynamic behaviour of the pathway is dominated by the rate of STAT de-phosphorylation and nuclear export and that these two variables result in bistable pathway behaviour when combined together in the model.

## Methods

### Narrative Modelling Language

For modelling the gp130/JAK/STAT pathway, we use the Narrative Language, a high-level textual language which has been recently designed for modelling dynamically regulated and spatially-confined biochemical pathways. Here we introduce the main features of the Narrative Language; for a detailed description of the language and of its translation into an executable language, the reader is referred to [10] and [27].

The basic entities of the language are molecules (*components*) and sub-cellular locations (*compartments*). In the language molecules can interact (e.g. bind/unbind), undergo biochemical modification (e.g. phosphorylation/de-phosphorylation) and re-locate between compartments. The time-dependent behaviour of the pathway is described in the form of a narrative of events involving these basic entities and functions, which imposes a temporal sequence and defines inter-dependencies and contingencies. In the narrative approach each of the elements can denote 'real' (i.e. experimentally defined) or abstract (e.g. hypothetical) entities. In silico exploration of the pathway model is simply enabled by modifying the narrative description and/or changing parameter values.

A feature of this narrative approach is the use of reliability values associated with numerical parameters. This is a percentage value which describes the reliability of the associated numerical value, and it can be used to distinguish between values that are highly certain because obtained from high quality biological experiment, and others which are inferred as a result of un-verified assumptions or 'guesswork' (e.g. *100% *indicates high precision data, while *0% *indicates a value which has no experimental evidence). Reliability values do not influence the behaviour of the programme but are annotations to inform use of the model. In particular modellers can employ reliability values to identify parameter dependencies to be explored during model refinement.

### Model description and simulation

The Narrative Language model of the gp130/JAK/STAT signalling pathway is supplied as Additional files 1, 2, 3, 4, 5, 6, 7 and 8. The molecular components we consider in the model are: two ligands (LIF and OSM), three membrane-bound receptors (gp130, LIFR and OSMR), one effector (STAT3), and two inhibitors (SOCS3 and PIAS3). The receptor associated kinase JAK and TC-PTP phosphatase are implicitly modelled.

Additional file 2 shows the definition of the components involved in the pathway. For each protein, the list of interaction/modification sites, states and locations are defined and initialised. Each receptor contains at least one ligand binding site (OSMR has only one site for OSM, while LIFR and gp130 also have one site for LIF), one binding site for SOCS3 inhibitor, and some phosphorylation sites. Moreover, receptors can be in dimeric state (an additional site in gp130 allows us to distinguish between the two types of OSM receptors). STAT3 has one phosphorylation and four binding sites (for receptors and PIAS3 inhibitor), and it can be monomeric or dimeric (STAT3 can form homodimers). The initial quantities are also defined. The number of ligands is calculated based on the known extracellular concentration (500 *pM*).

Four compartments are involved in the system (see additional file 1): the exosol (the extracellular space, where the ligands are located), the cell membrane (location of the receptors), the cytosol (initial location of the STAT3 effector), and the nucleus (to which the effector can translocate). The compartment volumes are calculated based on the average cell radius and ratio between intra-cellular compartment volumes stated in [28]. The number of spatial dimensions is used to distinguish between 2D compartments (i.e. membranes) and 3D ones.

Additional file 3 shows the definition of the biochemical reactions occurring in the pathway. Reaction types (e.g. binding, unbinding, phosphorylation, relocation) and rates (i.e. the kinetic constants) are defined here. The reaction volume is the volume in which the reaction occurs (generally it is the size of the compartment in which the involved species are located), and it is used in deriving the actual rate at which the reaction occurs. Some of the reaction rates have been obtained from wet experiments, while others have been estimated based on information about similar reactions, or extracted from other models [22-24]; reliability values are assigned to reaction rates and volumes.

Finally, Additional files 4, 5, 6, 7 and 8 show the definition of the narrative of events, which describes the evolution of the system; it is a sequence of basic events, each of which is a constrained textual description of a biochemical reaction involving at most two components. Moreover, events are grouped into processes for the sake of readability. The defined events describe the binding/unbinding of ligand/receptor pairs, the downstream LIF and OSM pathways (formation and activation of receptor complexes), the downstream STAT3 pathway (recruitment and activation of STAT3, and its nuclear/cytoplasmic shuttling), and the inhibition mechanisms.

Additional file 4 models the binding of ligands to receptors (reaction r1 in the graphical representation of the pathway shown in Figure 1 and events 1, 3, 5, 7 and 9 in the Narrative Language model), and the inverse unbinding reaction (r2, events 2, 4, 6, 8 and 10). For each event, the involved components and the occurring interaction are specified (e.g. event 1 states that LIF binds to receptor gp130 on a specific binding site) together with the activating conditions (e.g. for event 1, the binding site in gp130 is not already occupied, LIF itself is free, and gp130 is not already in dimeric form), and the reference to the corresponding reaction in Additional file 3.

Additional file 5 models the dimerisation of pairs of receptor subunits to form receptor complexes (gp130-LIFR or gp130-OSMR), which is triggered by the binding of a ligand to one of the receptors (r3, events 11, 13, 15, 17, 19 and 21) (e.g., in events 19 and 21, OSMR and gp130 form a heterodimeric complex if one of them has been previously activated by ligand binding); the dissociation of the receptor complexes is also modelled (r4, events 12, 14, 16, 18, 20 and 22).

Additional file 6 models the activation (JAK-mediated phosphorylation) of the receptor complexes, the binding of STAT3 to a receptor complex, and the activation (phosphorylation) of STAT3. Once the receptor dimeric complex is formed, each receptor subunit (gp130, LIFR and OSMR) can phosphorylate on specific sites (r5, events 23, 25, 27 and 29). STAT3 can bind on receptors' phosphorylated sites (r7, events 31, 32 and 33), and the binding of STAT3 allows the phosphorylation of STAT3 on site Y705 (r8, events 37, 38 and 39).

Additional file 7 models the unbinding of STAT3 from receptor complexes, its homodimerisation, and nuclear/cytoplasmic shuttling (relocation into the nucleus, de-phosphorylation by TC-PTP, de-homodimerisation and relocation into the cytoplasm). Once phosphorylated, STAT3 can dissociate from the receptor complex (r10, events 41, 42 and 43); the phosphorylated site allows STAT3 to homodimerise (r11, event 44). When STAT3 is in dimeric form, it can translocate into the nucleus (r12, event 45) where it can carry out its specific functions (not modelled here): STAT3 binds to the DNA, activating the transcription of downstream gene targets. Nuclear STAT3 is inactivated through de-phosphorylation by TC-PTP (r13, event 46), which leads to its de-dimerisation (r14, event 47), and its export to the cytoplasm (r15, event 48), where STAT3 can undergo additional cycles of activation.

Additional file 8 models SOCS3 and PIAS3 inhibition mechanisms. SOCS3 is produced by active STAT3 (r16, event 49) and degraded (event 50), and it acts by competing with STAT3 in binding to receptors (r17, events 51, 52 and 53). PIAS3 acts by binding to active nuclear STAT3 (r18, event 57).

We developed a tool [29], *N2BB*, which implements an automatic translation of models described in the biologically-intuitive Narrative Language into executable computable models formulated in *BlenX *[11], a programming language inspired on the *Beta-binders *process calculus [4]. Process calculi, originally developed for modelling mobile communicating systems, have recently been proposed as appropriate for simulating biological processes [1], and they have proved themselves as powerful tools for dynamical modelling of complex biological systems [5-8]. Differently from differential equations, process calculi also allow for analysis of models (e.g. model-checking, equivalence, reachability, causality, and locality analysis).

The BlenX model derived from the Narrative Language model is compatible with the BetaWB [25], a collection of tools for modelling, simulating, and analysing BlenX models. Hence, the model can be imported into the BetaWB designer, or directly simulated by means of the BetaWB simulator; the time-evolution of the simulation can be visualised by means of the BetaWB plotter or the Snazer tool [30]. For a detailed description of the BlenX language and of the implementation of the simulator, see [25,11].

### Cells, reagents and cytokines

MCF-7 human breast cancer cells were obtained from American Type Culture Collection (Manassas, VA) and cultured as described [13]. The human oncostatin M recombinant expression plasmid, pGEX-3C-OSM, was prepared, expressed and purified as described previously [13].

### Western blot, immunofluorescence and data analysis

Serum starved MCF-7 cells were stimulated with 10 ng/ml oncostatin M for increasing times (up to 480 minutes) at 37°C. For Western blot analysis, cell lysates were prepared and analysed as described [13] and monoclonal anti-phospho STAT3 (Tyr705) and STAT3 (Cell Signalling Technology) antibodies used for immunodetection. The density of the bands representing phospho-STAT3 and STAT3 were measured using ImageJ [31] and expressed as the ratio of phospho-STAT3 to STAT3. For immunofluorescence studies, MCF7 cells grown on coverslips were fixed with 4% paraformaldehyde (10 min, RT), permeabilised with 0.1% saponin solution (0.02 M glycine, 0.1% saponin, 0.1 M Tris/HCl pH8.5) for 20 min and blocked for 1 hour in 0.1% saponin solution (0.1% saponin, 0.1 M Tris/HCl pH8.5) plus 2.5% foetal calf serum. Cells were immunostained with monoclonal anti-STAT3 antibody for 1 h at RT and incubated with Texas Red-conjugated secondary antibody containing Hoechst (Molecular Probes) for 45 min at RT. Coverslips were mounted with 5 μl of Mowiol solution (10% Mowiol 4–88, 25% glycerol, 0.1 M Tris/HCl pH8.5) on the slide and observed under confocal microscope. For localisation analysis, images captured were converted to greyscale and total STAT3 fluorescence calculated from the sum of pixel density values using ImageJ. Nuclear STAT3 fluorescence was calculated from selection of nuclear area (as determined by Hoechst staining) and cytoplasmic STAT3 fluorescence calculated by subtracting nuclear staining from total cellular staining. For each time-point analysis was performed on between 60 – 100 cells (from multiple coverslips) and mean values ± 2 SD were calculated for total nuclear and cytoplasmic STAT (expressed as percentage of total STAT).

## Results and discussion

We used the N2BB tool to automatically translate the gp130/JAK/STAT pathway model into BlenX and we simulated the derived model using the BetaWB. Our intention in the simulations that follow was to firstly define the behaviour of the full model and then to study the behaviour of the model in response to perturbations such as modifying numerical values and/or removing components from the model. A particular feature of the narrative approach is that it is very simple to change parameters or modify the narrative of events in the process of model exploration.

Our aim is to define those features, which exert the dominating influence on the dynamic behaviour of the pathway in the model for evaluation by biological experiment. This is an example of using computer models to explore a wide range of scenarios in silico to guide the formulation of the more laborious and expensive laboratory-based experiments.

**Figure 2:**
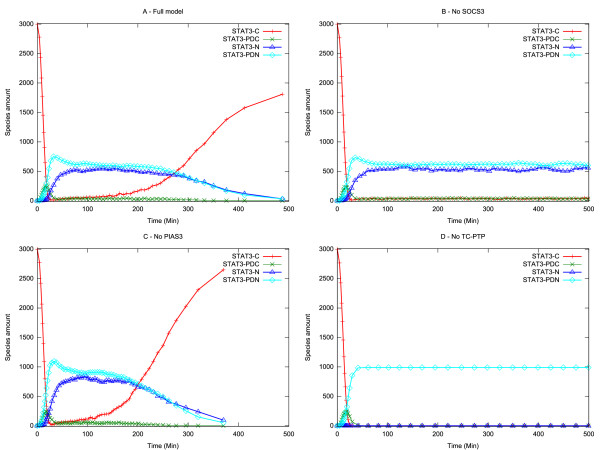
**Role of inhibitors in the gp130/JAK/STAT pathway**. (A) Full model. (B) SOCS3 knock out. (C) PIAS3 knock out. (D) TC-PTP knock out. For each experiment the amounts of different STAT3 forms are plotted: monomeric cytoplasmic (STAT3-C), dimeric cytoplasmic (STAT3-PDC), monomeric nuclear (STAT3-N), and dimeric nuclear (STAT3-PDN).

The time-evolution resulting from the simulation of the model is shown in the following pictures. Figure 2A reports the time-evolution of the full model, while Figure 2B–2D show the evolution when different inhibitors are removed (in silico genetics). The amounts of different STAT3 forms are plotted: monomeric cytoplasmic, dimeric cytoplasmic, monomeric nuclear, and dimeric nuclear.

Figure 2A shows that in the initial configuration STAT3 is present in monomeric form in the cytoplasm. As the system undergoes dynamic evolution, STAT3 is rapidly phosphorylated, dimerised and translocated into the nucleus. At the same time cytoplasmic STAT3 is dramatically reduced. The system reaches a plateau for some time and then slowly reverses as nuclear STAT3 levels fall and cytoplasmic levels rise. This behaviour conforms well to that obtained experimentally by observing the time-evolution of STAT3 phosphorylation (Additional file 9) and the relocation of STAT3 to the nuclear compartment in response to gp130 activation (Additional file 10). However it is important to note that, whilst the model and experimental data are in quantitative agreement for early time points (0–200 minutes), the experimental data reveals a faster rate of recovery of cytoplasmic STAT3 than predicted from the model over longer time periods. We were unable to accelerate the rate of relocalisation of STAT3 in the model by parameter variation (data not shown) which we interpret as indicating that the current model does not include biological processes that influence the rate of nuclear/cytoplasmic shuttling at later time points. In this context we remark that the current model takes no account of the induction of new gene expression [13] by gp130 signalling over this time period which could include components that influence the rate of nuclear/cytoplasmic transfer.

We conclude that the computer model derived from the original narrative is able to capture the dynamic behaviour of the real pathway, demonstrating the validity of the approach.

In the next phase we explored the dependency of the model on the presence of various components which were 'knocked out' by removal from the programme.

By comparing Figure 2A and 2B, in which we run the simulation in the absence of SOCS3, we observe that the effect of SOCS3 expression in response to STAT3 activation is to activate the slow attenuation process observed in the full model: removal of SOCS3 suppresses the delayed re-appearance of cytoplasmic STAT3 and the pathway exhibits prolonged and stable nuclear occupancy of STAT3.

The role PIAS3, which binds to phosphorylated nuclear STAT3 preventing it binding to DNA, is revealed by comparing Figure 2A and 2C. In this case removal of PIAS3 yields an initial increase of active STAT3, but the nuclear STAT3 signal attenuates with a faster time-course than in the full model, leading to an increase in cytoplasmic (and therefore inactive) STAT3.

Figure 2D shows the outcomes of removing the nuclear phosphatase TC-PTP from the model. In this case there is a rapid accumulation of nuclear STAT3 which reaches steady state and fails to attenuate. In this case cytoplasmic STAT3 is rapidly eliminated and does not re-appear.

Comparing the consequences of removing three different types of inhibitor from the model it is clear that each has characteristic temporal effects. The consequences of TC-PTP inhibition are significantly more rapid than removal of SOCS3 or PIAS3. Inhibition of SOCS3 and TC-PTP lead to prolonged and stable activated STAT3, whereas inhibition of PIAS3 accelerates the rate of activated STAT3 decay by inducing accumulation of cytoplasmic STAT3.

### Single parameter sensitivity analysis

We next turned to analysis of the parameter sensitivities of the model. In this case we ran simulations in which individual parameters were systematically varied to observe the dependency of model behaviour on individual values. The aim here is to define those values, which have greatest impact on model behaviour.

**Figure 3:**
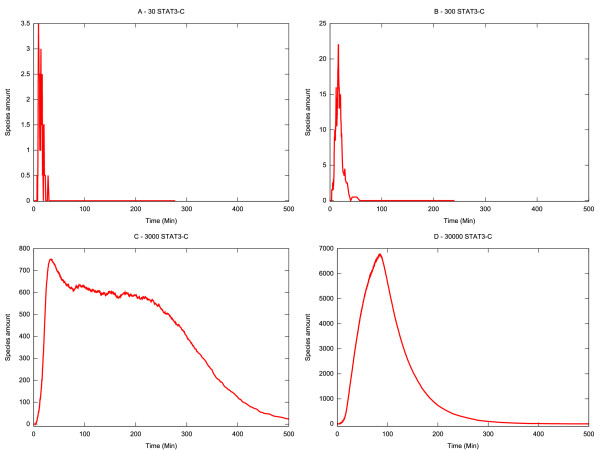
**Single parameter sensitivity analysis: variation of STAT3 initial amount**. The evolution of the system has been observed varying the initial amount of STAT3. (A) 30 molecules. (B) 300 molecules. (C) 3000 molecules. (D) 30000 molecules. The amount of active nuclear STAT3 (STAT3-PDN) is plotted.

We first examined the dependency of the model on the amount of STAT3 in the system. In this case we ran simulations containing different numbers of STAT3 molecules at initialisation (Figure 3). We observe that the duration of nuclear occupancy is relatively stable to STAT3 perturbation until it drops below a threshold value (in this case between 30–300 molecules) when it collapses into 'noise'. However the number of STAT3 molecules had a significant effect on peak amplitude where there was an approximately linear relationship between the maximum number of activated STAT3 molecules reached and the total number of molecules in the system.

**Figure 4:**
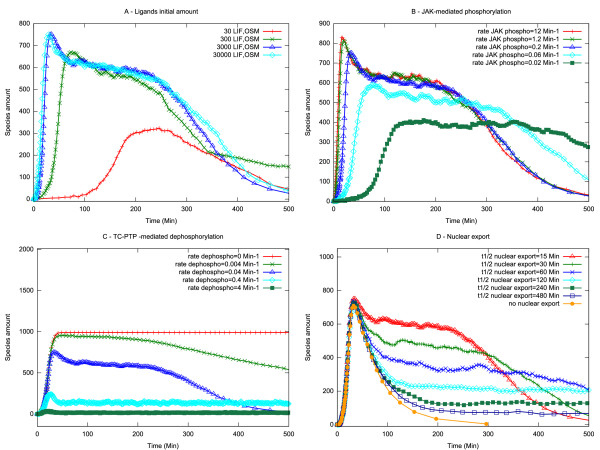
**Single parameter sensitivity analysis**. The evolution of the system has been observed varying each parameter individually. (A) Ligands initial amount. (B) JAK-mediated phosphorylation. (C) TC-PTP-mediated de-phosphorylation. (D) Nuclear export. The amount of active nuclear STAT3 (STAT3-PDN) is plotted.

We next examined the rate of TC-PTP de-phosphorylation, the amount of ligand, the rate of JAK kinase phosphorylation and the rate of nuclear export (Figure 4A–4D). The outcomes of these simulations show that the behaviour of the model is differentially dependent on particular values.

Slowing the rate of phospho-STAT3 de-phosphorylation exhibited significant effects on activated STAT3 amplitude and duration noted over the complete range analysed (Figure 4C). De-phosphorylation rates impacted on both the peak amplitude and duration of activated STAT3 indicating, as reported by others [32], that nuclear de-phosphorylation of activated STAT3 is an important determinant of signalling dynamics. Similar sensitivities were found on varying the rate of nuclear export (Figure 4D): although in this case nuclear export had no impact on peak signal amplitude and its main effect was on signal duration.

By contrast the model was relatively robust to variations in either the amount of ligand in the system or the rate of JAK activation (Figure 4A and 4B) where significant impacts on signal dynamics only become apparent at extreme values. Indeed, similar to the dependency on STAT3 numbers, it appears as though behaviour of the model is relatively robust to parameter changes in these processes over several orders of magnitude.

### Multi-dimensional parameter sensitivity

**Figure 5:**
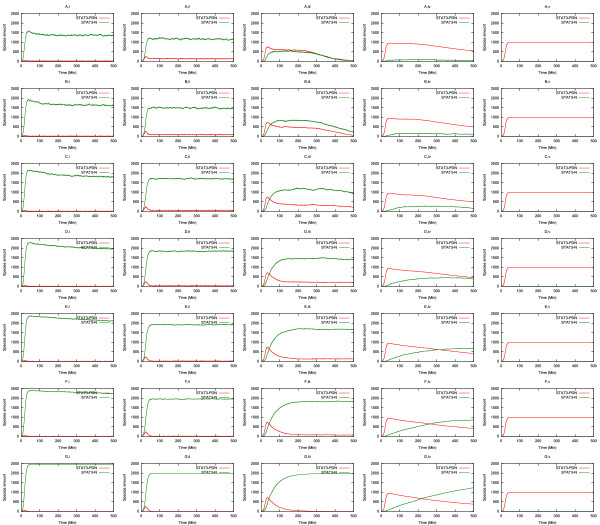
**Multi-dimensional parameter sensitivity**. The evolution of the system has been observed varying simultaneously the rate of nuclear STAT3 de-phosphorylation and the rate of STAT3 nuclear export. The considered values are as in Figure 4C and 4D. The rows (A-G) refer to the export rate, while the columns (i-v) refer to the de-phosphorylation rate. The amounts of active nuclear STAT3 (STAT3-PDN) and monomeric nuclear STAT3 (STAT3-N) are plotted.

The foregoing in silico experiments revealed that the dynamic behaviour of the gp130/JAK/STAT pathway is most sensitive to two parameters: the rate of nuclear STAT3 de-phosphorylation and the rate of STAT3 nuclear export. We were interested to learn if the model exhibited higher order dependencies when parameters were varied in combinations. Exhaustive implementation of this approach is currently computationally expensive. For this study we therefore chose to study the interaction between nuclear de-phosphorylation of STAT3 and export of de-phosphorylated STAT3. To this end 35 simulations were run in which each parameter was changed simultaneously (Figure 5). The results of this experiment were surprising. Instead of exhibiting a graded response across the whole parameter landscape, as might be predicted from the behaviour of each individual value, the system exhibits a bistable response in which, for the majority of conditions, the system is either constitutively activated or constitutively repressed. The system only exhibits sensitivity to parameter variation in a narrow region of values towards the middle of the ranges chosen. In this region both peak amplitude and signal duration were dependant upon the interplay between nuclear export and STAT3 de-phosphorylation.

## Conclusion

Our purpose in this study was to explore the practical utility of the biological narrative approach for in silico exploration of a complex signalling pathway. We used the gp130/JAK/STAT pathway as a case study, and we showed that the biologist-specified narrative yields outcomes which conform well to experimental data. By 'experimenting' on the model by parameter exploration and component removal we were able to explore the influence of different elements on the dynamic behaviour of the pathway. Some of these experiments had already been performed in previous works [22-24], and the results we obtained show a good agreement with them; others provide novel insight on the pathway behaviour. Our analyses showed that the rate of STAT nuclear export and nuclear localised de-phosphorylation were key determinants of signalling dynamics. This conclusion is supported by in vitro experimental data in which inhibition of nuclear export by either drug treatment [33,34] or mutation of the Nuclear Export Sequence [35] results in prolonged nuclear accumulation of phosphorylated STAT. The dependency of STAT import/export is also in agreement with existing mathematical models of the same pathway [22-24].

This outcome indicates that the model captures the dynamic behaviour of the real pathway well and encourages further exploration of the model into issues which would be resource intensive – or technically challenging – to address by biological experimentation. In particular we were interested in exploring the potential for interactions between parameters which are not currently addressable by biological experiment. We found, combining nuclear export and nuclear de-phosphorylation, that the two parameters interact strongly yielding a 'switch-like' behaviour. This type of modelling analysis may inform future considerations of multi-step mutagenesis or combination drug therapy scenarios in the gp130/JAK/STAT pathway.

The identified interaction between nuclear export and nuclear de-phosphorylation is novel, and the obtained behaviour was unpredicted; the insensitivity to the rate of JAK phosphorylation is another interesting outcome. These novel results reinforce our belief about the predictive power of this kind of modelling approach. In order to be validated, the hypotheses deriving from our simulation results should be tested by means of specific laboratory experiments. For instance, the characteristic dynamic shifts exhibited when the three inhibitors are removed from the system could be tested using appropriate mutant cells or by the use of specific pharmacological inhibitors. However, in general this might not be a simple task because it often involves the generation of such cells/drugs.

We have demonstrated in this study that a language can be used to describe biological signalling pathways in a way, which is formal and unambiguous for computational execution but more intuitive to the biologist compared with standard modelling languages. The approach exploits the particular advantages of the "molecule as computation" paradigm of Regev and Shapiro [1]: the resulting models are computable, relevant and understandable. There are also practical advantages to process calculus models in that they can be readily modified to explore different scenarios and interrogated using model-checking tools [3] to formally verify the model and explore its quantitative behaviour. Collectively the approach is therefore extensible in that, as new biological information on the pathway becomes available, it can be readily incorporated into the model.

In addition to the advantage of being simpler to use for modelling compared to directly specifying models in formal languages such as process calculi, the Narrative Language also provides modellers with a number of explicit features which capture some cardinal aspects of biological signalling pathways. It defines the temporal relationships between events (i.e. sequential, concurrent and competing events). It defines the location of proteins and the reaction volumes, and it deals with multi-compartmental models thereby making spatial location and confinement a central feature. Species in the model can exist in multiple states and locations. The Narrative Language can therefore be employed to model any biological process involving state transitions of different types; inter-molecular interactions; spatial confinement and temporal evolution. In order to assess the generalisability of the approach to model other biochemical systems, we have analysed a number of simple standard models using our and other existing simulation approaches and we have observed a good agreement between the results (results not reported here). Moreover, the biochemical entities, interactions and information which can be modelled in the Narrative Language are very similar to the ones modelled in the representation used in the NCI-Nature Pathway Interaction Database (PID) [36], a curated collection of biomolecular pathways represented in a graphical language. Considered the analogies between the PID and our proposal, we are currently developing a mapping between the PID representation and the Narrative Language. This translation would provide us with a significant number of well-understood pathways which can be directly simulated.

Finally the simulation results shown in this paper are obtained using N2BB, which translates Narrative Language models into BlenX models, but the translation of the Narrative Language into other current or future languages is possible. Thus the biological formulation of the pathway is separable from the computer method employed for simulation and analysis, allowing for interoperability with other tools for modelling and analysis of biochemical systems. For instance, a translation into the Bio-PEPA process algebra [37] is currently under development, and would allow modellers to describe models in the Narrative Language and study them by means of the various analysis methods usable for Bio-PEPA models (e.g. model-checking and mathematical techniques based on differential equations).

## Authors' contributions

MLG designed the computational model, performed the simulation experiments, analysed the data and wrote the paper. AD performed the laboratory experiments and analysed the data. NUD participated in designing the model, performed the laboratory experiments and analysed the data. JKH participated in designing the model, analysed the data and wrote the paper. CP participated in analysing the data and writing the paper. All authors participated in conceiving the experiments, read and approved the final manuscript.

## Supplementary Material

Additional file 1 **Table 1**. Gp130/JAK/STAT pathway model: list of compartments. Click here for file

Additional file 2 **Table 2**. Gp130/JAK/STAT pathway model: list of components. Click here for file

Additional file 3 **Table 3**. Gp130/JAK/STAT pathway model: list of reactions. Click here for file

Additional file 4 **Table 4**. Gp130/JAK/STAT pathway model: list of events (ligand-receptor bindings). Click here for file

Additional file 5 **Table 5**. Gp130/JAK/STAT pathway model: list of events (receptor complexes formation). Click here for file

Additional file 6 **Table 6**. Gp130/JAK/STAT pathway model: list of events (STAT3 activation). Click here for file

Additional file 7 **Table 7**. Gp130/JAK/STAT pathway model: list of events (STAT3 unbinding and shuttling). Click here for file

Additional file 8 **Table 8**. Gp130/JAK/STAT pathway model: list of events (SOCS3 and PIAS3 inhibition). Click here for file

Additional file 9 **Time-evolution of STAT3 phosphorylation**. (A) Levels of phospho-STAT3 (upper panel) and total STAT3 (lower panel) after stimulation with oncostatin M. (B) Densitometric analysis of immunoblots showing the time-course of phospho-STAT3/STAT3 ratios. (C) Simulation time-course of phospho-STAT3/STAT3 ratios. Click here for file

Additional file 10 **Nuclear localisation of STAT3**. (A) Time-course of STAT3 nuclear/cytoplasmic localisation. (B) Images representative of STAT3 localisation after stimulation with Oncostatin M: (i) control; (ii) 20 minutes; (iii) 4 hours; (iv) 8 hours. (C) Simulation time-course of STAT3 nuclear/cytoplasmic localisation. Click here for file
