# Supplementary material for: Narrative-based computational modelling of the Gp130/JAK/STAT signalling pathway
Source: BMC Syst Biol. 2009 Apr 15;3:40. doi: 10.1186/1752-0509-3-40 (PMC2678071; doi:10.1186/1752-0509-3-40)
Supplement: Additional file 1 — Table 1. Gp130/JAK/STAT pathway model: list of compartments. [file 1752-0509-3-40-S1.pdf]

| id | name | size | unit of measure | dimensions |
| --- | --- | --- | --- | --- |
| 1 | **exosol** | $9.91 \times 10^{-12}$ | L | 3 |
| 2 | **cellMembrane** | $1.26 \times 10^{-7}$ | $dm^2$ | 2 |
| 3 | **cytosol** | $2.09 \times 10^{-12}$ | L | 3 |
| 4 | **nucleus** | $0.25 \times 10^{-12}$ | L | 3 |
