# Supplementary material for: Narrative-based computational modelling of the Gp130/JAK/STAT signalling pathway
Source: BMC Syst Biol. 2009 Apr 15;3:40. doi: 10.1186/1752-0509-3-40 (PMC2678071; doi:10.1186/1752-0509-3-40)
Supplement: Additional file 2 — Table 2. Gp130/JAK/STAT pathway model: list of components. [file 1752-0509-3-40-S2.pdf]

name	descr	site	site_state	site_act	state	state_act	comp	comp_act	init_amount	rel
LIF	ligand				**bound**	**false**	1	**true**	3000	100%
OSM	ligand				**bound**	**false**	1	**true**	3000	100%
gp130	receptor	LIF	**bound**	**false**	**bound**	**false**	2	**true**	1000	50%
		OSM	**bound**	**false**						
		Y767	**phospho**	**false**						
		Y814	**phospho**	**false**						
		Y905	**phospho**	**false**						
		Y915	**phospho**	**false**						
		SOCS3	**bound**	**false**						
		typeI	**dimer**	**false**						
		typeII	**dimer**	**false**						
LIFR	receptor	LIF	**bound**	**false**	**bound**	**false**	2	**true**	1000	50%
		OSM	**bound**	**false**	**dimer**	**false**				
		Y981	**phospho**	**false**						
		Y1001	**phospho**	**false**						
		Y1028	**phospho**	**false**						
		SOCS3	**bound**	**false**						
OSMR	receptor	OSM	**bound**	**false**	**bound**	**false**	2	**true**	1000	50%
		Y917	**phospho**	**false**	**dimer**	**false**				
		Y945	**phospho**	**false**						
		SOCS3	**bound**	**false**						
STAT3	effector	Y705	**phospho**	**false**	**dimer**	**false**	3	**true**	3000	30%
		gp130	**bound**	**false**			4	**false**		
		LIFR	**bound**	**false**						
		OSMR	**bound**	**false**						
		PIAS3	**bound**	**false**						
SOCS3	inhibitor				**bound**	**false**	3	**true**	0	100%
PIAS3	inhibitor				**bound**	**false**	4	**true**	1000	20%
