# Supplementary material for: Narrative-based computational modelling of the Gp130/JAK/STAT signalling pathway
Source: BMC Syst Biol. 2009 Apr 15;3:40. doi: 10.1186/1752-0509-3-40 (PMC2678071; doi:10.1186/1752-0509-3-40)
Supplement: Additional file 3 — Table 3. Gp130/JAK/STAT pathway model: list of reactions. [file 1752-0509-3-40-S3.pdf]

id	type	rate	unit	rel	react_vol	unit	rel
1	**binding**	4.8×10^{7}	$M^{-1}Min^{-1}$	80%	$9.91\cdot10^{-12}$	l	50%
2	**unbinding**	3.6×10^{-1}	Min^{-1}	80%	$9.91\cdot10^{-12}$	l	50%
3	**binding**	4.8×10^{7}	$M^{-1}Min^{-1}$	80%	$9.91\cdot10^{-12}$	l	50%
4	**unbinding**	3.6×10^{-2}	Min^{-1}	80%	$9.91\cdot10^{-12}$	l	50%
5	**binding**	4.8×10^{7}	$M^{-1}Min^{-1}$	80%	$9.91\cdot10^{-12}$	l	50%
6	**unbinding**	3.6×10^{-2}	Min^{-1}	80%	$9.91\cdot10^{-12}$	l	50%
7	**binding**	4.8×10^{7}	$M^{-1}Min^{-1}$	80%	$9.91\cdot10^{-12}$	l	50%
8	**unbinding**	3.6×10^{-1}	Min^{-1}	80%	$9.91\cdot10^{-12}$	l	50%
9	**binding**	4.8×10^{7}	$M^{-1}Min^{-1}$	80%	$9.91\cdot10^{-12}$	l	50%
10	**unbinding**	3.6×10^{-2}	Min^{-1}	80%	$9.91\cdot10^{-12}$	l	50%
11	**dimerization**	**inf**	$M^{-1}Min^{-1}$	50%	$9.91\cdot10^{-12}$	l	50%
12	**dedimerization**	0	$M^{-1}Min^{-1}$	0%	$9.91\cdot10^{-12}$	l	50%
13	**phosphorylation**	0.2	Min^{-1}	80%	$9.91\cdot10^{-12}$	l	50%
14	**dephosphorylation**	0	Min^{-1}	0%	$9.91\cdot10^{-12}$	l	50%
15	**binding**	4.8×10^{8}	$M^{-1}Min^{-1}$	20%	$2.09\cdot10^{-12}$	l	50%
16	**unbinding**	0.06	Min^{-1}	30%	$2.09\cdot10^{-12}$	l	50%
17	**phosphorylation**	0.2	Min^{-1}	80%	$2.09\cdot10^{-12}$	l	50%
18	**dephosphorylation**	0	Min^{-1}	0%	$2.09\cdot10^{-12}$	l	50%
19	**unbinding**	**inf**	Min^{-1}	10%	$2.09\cdot10^{-12}$	l	50%
20	**homodimerization**	**inf**	Min^{-1}	50%	$2.09\cdot10^{-12}$	l	50%
21	**relocation**	1	Min ($t_{1/2}$)	10%	$2.09\cdot10^{-12}$	l	50%
22	**dephosphorylation**	0.04	Min^{-1}	20%	$0.25\cdot10^{-12}$	l	50%
23	**dehomodimerization**	**inf**	Min^{-1}	20%	$0.25\cdot10^{-12}$	l	50%
24	**relocation**	15	Min ($t_{1/2}$)	10%	$0.25\cdot10^{-12}$	l	50%
25	**synthesis**	0.01	Min^{-1}	50%	$0.25\cdot10^{-12}$	l	50%
26	**binding**	6.0×10^{7}	$M^{-1}Min^{-1}$	20%	$2.09\cdot10^{-12}$	l	50%
27	**unbinding**	0.006	Min^{-1}	30%	$2.09\cdot10^{-12}$	l	50%
28	**binding**	1.0×10^{8}	$M^{-1}Min^{-1}$	20%	$0.25\cdot10^{-12}$	l	50%
29	**unbinding**	0.06	Min^{-1}	30%	$0.25\cdot10^{-12}$	l	50%
30	**degradation**	0.01	Min^{-1}	50%	$0.25\cdot10^{-12}$	l	50%
