# Supplementary material for: Narrative-based computational modelling of the Gp130/JAK/STAT signalling pathway
Source: BMC Syst Biol. 2009 Apr 15;3:40. doi: 10.1186/1752-0509-3-40 (PMC2678071; doi:10.1186/1752-0509-3-40)
Supplement: Additional file 4 — Table 4. Gp130/JAK/STAT pathway model: list of events (ligand-receptor bindings). [file 1752-0509-3-40-S4.pdf]

id	description	react	alt
	LIF-gp130 binding		
1	**if** gp130.LIF **is not bound and** LIF **is not bound and** gp130.typeI **is not dimer and** gp130.typeII **is not dimer then** LIF **binds** gp130 **on** LIF	1	
2	**if** gp130.LIF **is bound and** LIF **is bound and** gp130.typeI **is not dimer and** gp130.typeII **is not dimer and** gp130.Y767 **is not phospho then** LIF **unbinds** gp130 **on** LIF	2	
	LIF-LIFR binding		
3	**if** LIFR.LIF **is not bound and** LIF **is not bound and** LIFR **is not dimer then** LIF **binds** LIFR **on** LIF	3	
4	**if** LIFR.LIF **is bound and** LIF **is bound and** LIFR **is not dimer and** LIFR.Y981 **is not phospho then** LIF **unbinds** LIFR **on** LIF	4	
	OSM-gp130 binding		
5	**if** gp130.OSM **is not bound and** OSM **is not bound and** gp130.typeI **is not dimer and** gp130.typeII **is not dimer then** OSM **binds** gp130 **on** OSM	5	1
6	**if** gp130.OSM **is bound and** OSM **is bound and** gp130.typeI **is not dimer and** gp130.typeII **is not dimer and** gp130.Y767 **is not phospho then** OSM **unbinds** gp130 **on** OSM	6	
	OSM-LIFR binding		
7	**if** LIFR.OSM **is not bound and** OSM **is not bound and** LIFR **is not dimer then** OSM **binds** LIFR **on** OSM	7	3
8	**if** LIFR.OSM **is bound and** OSM **is bound and** LIFR **is not dimer and** LIFR.Y981 **is not phospho then** OSM **unbinds** LIFR **on** OSM	8	
	OSM-OSMR binding		
9	**if** OSMR.OSM **is not bound and** OSM **is not bound and** OSMR **is not dimer then** OSM **binds** OSMR **on** OSM	9	
10	**if** OSMR.OSM **is bound and** OSM **is bound and** OSMR **is not dimer and** OSMR.Y917 **is not phospho then** OSM **unbinds** OSMR **on** OSM	10	
