# Supplementary material for: Narrative-based computational modelling of the Gp130/JAK/STAT signalling pathway
Source: BMC Syst Biol. 2009 Apr 15;3:40. doi: 10.1186/1752-0509-3-40 (PMC2678071; doi:10.1186/1752-0509-3-40)
Supplement: Additional file 5 — Table 5. Gp130/JAK/STAT pathway model: list of events (receptor complexes formation). [file 1752-0509-3-40-S5.pdf]

id	description	react	alt
	LIF pathway → type I receptor gp130:LIFR		
11	**if** gp130.LIF **is bound and** LIFR.LIF **is not bound and** gp130.OSM **is not bound and** LIFR.OSM **is not bound and** LIFR **is not dimer and** gp130.typeI **is not dimer and** gp130.typeII **is not dimer then** LIFR **dimerizes with** gp130 **on** typeI	11	2
12	**if** gp130.LIF **is bound and** LIFR.LIF **is not bound and** gp130.OSM **is not bound and** LIFR.OSM **is not bound and** LIFR **is dimer and** gp130.typeI **is dimer and** gp130.typeII **is not dimer and** LIFR.Y981 **is not phospho and** gp130.Y767 **is not phospho then** LIFR **dedimerizes from** gp130 **on** typeI	12	
13	**if** LIFR.LIF **is bound and** gp130.LIF **is not bound and** LIFR.OSM **is not bound and** gp130.OSM **is not bound and** LIFR **is not dimer and** gp130.typeI **is not dimer and** gp130.typeII **is not dimer then** LIFR **dimerizes with** gp130 **on** typeI	11	4
14	**if** LIFR.LIF **is bound and** gp130.LIF **is not bound and** LIFR.OSM **is not bound and** gp130.OSM **is not bound and** LIFR **is dimer and** gp130.typeI **is dimer and** gp130.typeII **is not dimer and** LIFR.Y981 **is not phospho and** gp130.Y767 **is not phospho then** LIFR **dedimerizes from** gp130 **on** typeI	12	
	OSM pathway → type I receptor gp130:LIFR		
15	**if** gp130.OSM **is bound and** LIFR.OSM **is not bound and** gp130.LIF **is not bound and** LIFR.LIF **is not bound and** LIFR **is not dimer and** gp130.typeI **is not dimer and** gp130.typeII **is not dimer then** LIFR **dimerizes with** gp130 **on** typeI	11	6
16	**if** gp130.OSM **is bound and** LIFR.OSM **is not bound and** gp130.LIF **is not bound and** LIFR.LIF **is not bound and** LIFR **is dimer and** gp130.typeI **is dimer and** gp130.typeII **is not dimer and** LIFR.Y981 **is not phospho and** gp130.Y767 **is not phospho then** LIFR **dedimerizes from** gp130 **on** typeI	12	
17	**if** LIFR.OSM **is bound and** gp130.OSM **is not bound and** LIFR.LIF **is not bound and** gp130.LIF **is not bound and** LIFR **is not dimer and** gp130.typeI **is not dimer and** gp130.typeII **is not dimer then** LIFR **dimerizes with** gp130 **on** typeI	11	8
18	**if** LIFR.OSM **is bound and** gp130.OSM **is not bound and** LIFR.LIF **is not bound and** gp130.LIF **is not bound and** LIFR **is dimer and** gp130.typeI **is dimer and** gp130.typeII **is not dimer and** LIFR.Y981 **is not phospho and** gp130.Y767 **is not phospho then** LIFR **dedimerizes from** gp130 **on** typeI	12	
	OSM pathway → type II receptor gp130:OSMR		
19	**if** gp130.OSM **is bound and** OSMR.OSM **is not bound and** gp130.LIF **is not bound and** OSMR.LIF **is not bound and** OSMR **is not dimer and** gp130.typeI **is not dimer and** gp130.typeII **is not dimer then** OSMR **dimerizes with** gp130 **on** typeII	11	6
20	**if** gp130.OSM **is bound and** OSMR.OSM **is not bound and** gp130.LIF **is not bound and** OSMR.LIF **is not bound and** OSMR **is dimer and** gp130.typeI **is not dimer and** gp130.typeII **is dimer and** OSMR.Y917 **is not phospho and** gp130.Y767 **is not phospho then** OSMR **dedimerizes from** gp130 **on** typeII	12	
21	**if** OSMR.OSM **is bound and** gp130.OSM **is not bound and** OSMR.LIF **is not bound and** gp130.LIF **is not bound and** OSMR **is not dimer and** gp130.typeI **is not dimer and** gp130.typeII **is not dimer then** OSMR **dimerizes with** gp130 **on** typeII	11	10
22	**if** OSMR.OSM **is bound and** gp130.OSM **is not bound and** OSMR.LIF **is not bound and** gp130.LIF **is not bound and** OSMR **is dimer and** gp130.typeI **is not dimer and** gp130.typeII **is dimer and** OSMR.Y917 **is not phospho and** gp130.Y767 **is not phospho then** OSMR **dedimerizes from** gp130 **on** typeII	12	
