# Supplementary material for: Narrative-based computational modelling of the Gp130/JAK/STAT signalling pathway
Source: BMC Syst Biol. 2009 Apr 15;3:40. doi: 10.1186/1752-0509-3-40 (PMC2678071; doi:10.1186/1752-0509-3-40)
Supplement: Additional file 6 — Table 6. Gp130/JAK/STAT pathway model: list of events (STAT3 activation). [file 1752-0509-3-40-S6.pdf]

id	description	react	alt
	Receptors phosphorylation		
23	**if** gp130.typeI **is dimer and** gp130 **is not bound then** gp130 **phospho on** Y767;Y814;Y905;Y915	13	
24	**if** gp130.typeI **is dimer and** gp130 **is not bound then** gp130 **dephospho on** Y767;Y814;Y905;Y915	14	
25	**if** LIFR **is dimer and** LIFR **is not bound then** LIFR **phospho on** Y981;Y1001;Y1028	13	
26	**if** LIFR **is dimer and** LIFR **is not bound then** LIFR **dephospho on** Y981;Y1001;Y1028	14	
27	**if** gp130.typeII **is dimer and** gp130 **is not bound then** gp130 **phospho on** Y767;Y814;Y905;Y915	13	
28	**if** gp130.typeII **is dimer and** gp130 **is not bound then** gp130 **dephospho on** Y767;Y814;Y905;Y915	14	
29	**if** OSMR **is dimer and** OSMR **is not bound then** OSMR **phospho on** Y917;Y945	13	
30	**if** OSMR **is dimer and** OSMR **is not bound then** OSMR **dephospho on** Y917;Y945	14	
	STAT3 binding		
31	**if** gp130.Y767 **is phospho and** STAT3 **is in** 3 **and** STAT3 **is not dimer and** gp130 **is not bound and** gp130.SOCS3 **is not bound and** STAT3.gp130 **is not bound and** STAT3.LIFR **is not bound and** STAT3.OSMR **is not bound and** STAT3.Y705 **is not phospho then** gp130 **binds** STAT3 **on** gp130	15	
32	**if** LIFR.Y981 **is phospho and** STAT3 **is in** 3 **and** STAT3 **is not dimer and** LIFR **is not bound and** LIFR.SOCS3 **is not bound and** STAT3.LIFR **is not bound and** STAT3.gp130 **is not bound and** STAT3.OSMR **is not bound and** STAT3.Y705 **is not phospho then** LIFR **binds** STAT3 **on** LIFR	15	
33	**if** OSMR.Y917 **is phospho and** STAT3 **is in** 3 **and** STAT3 **is not dimer and** OSMR **is not bound and** OSMR.SOCS3 **is not bound and** STAT3.OSMR **is not bound and** STAT3.gp130 **is not bound and** STAT3.LIFR **is not bound and** STAT3.Y705 **is not phospho then** OSMR **binds** STAT3 **on** OSMR	15	
34	**if** gp130.Y767 **is phospho and** STAT3 **is in** 3 **and** STAT3 **is not dimer and** gp130 **is bound and** gp130.SOCS3 **is not bound and** STAT3.gp130 **is bound and** STAT3.LIFR **is not bound and** STAT3.OSMR **is not bound and** STAT3.Y705 **is not phospho then** gp130 **unbinds** STAT3 **on** gp130	16	
35	**if** LIFR.Y981 **is phospho and** STAT3 **is in** 3 **and** STAT3 **is not dimer and** LIFR **is bound and** LIFR.SOCS3 **is not bound and** STAT3.LIFR **is bound and** STAT3.gp130 **is not bound and** STAT3.OSMR **is not bound and** STAT3.Y705 **is not phospho then** LIFR **unbinds** STAT3 **on** LIFR	16	
36	**if** OSMR.Y917 **is phospho and** STAT3 **is in** 3 **and** STAT3 **is not dimer and** OSMR **is bound and** OSMR.SOCS3 **is not bound and** STAT3.OSMR **is bound and** STAT3.gp130 **is not bound and** STAT3.LIFR **is not bound and** STAT3.Y705 **is not phospho then** OSMR **unbinds** STAT3 **on** OSMR	16	
	STAT3 phosphorylation		
37	**if** STAT3.gp130 **is bound then** STAT3 **phosphorylates on** Y705	17	
38	**if** STAT3.LIFR **is bound then** STAT3 **phosphorylates on** Y705	17	
39	**if** STAT3.OSMR **is bound then** STAT3 **phosphorylates on** Y705	17	
40	**if** STAT3 **is in** 3 **then** STAT3 **dephosphorylates on** Y705	18	
