# Supplementary material for: Narrative-based computational modelling of the Gp130/JAK/STAT signalling pathway
Source: BMC Syst Biol. 2009 Apr 15;3:40. doi: 10.1186/1752-0509-3-40 (PMC2678071; doi:10.1186/1752-0509-3-40)
Supplement: Additional file 7 — Table 7. Gp130/JAK/STAT pathway model: list of events (STAT3 unbinding and shuttling). [file 1752-0509-3-40-S7.pdf]

id	description	react	alt
	STAT3 unbinding and homodimerization		
41	**if** gp130.Y767 **is phospho and** STAT3 **is in** 3 **and** STAT3 **is not dimer and** gp130 **is bound and** gp130.SOCS3 **is not bound and** STAT3.gp130 **is bound and** STAT3.LIFR **is not bound and** STAT3.OSMR **is not bound and** STAT3.Y705 **is phosphorylated then** gp130 **unbinds** STAT3 **on** gp130	19	
42	**if** LIFR.Y981 **is phospho and** STAT3 **is in** 3 **and** STAT3 **is not dimer and** LIFR **is bound and** LIFR.SOCS3 **is not bound and** STAT3.LIFR **is bound and** STAT3.gp130 **is not bound and** STAT3.OSMR **is not bound and** STAT3.Y705 **is phosphorylated then** LIFR **unbinds** STAT3 **on** LIFR	19	
43	**if** OSMR.Y917 **is phospho and** STAT3 **is in** 3 **and** STAT3 **is not dimer and** OSMR **is bound and** OSMR.SOCS3 **is not bound and** STAT3.OSMR **is bound and** STAT3.gp130 **is not bound and** STAT3.LIFR **is not bound and** STAT3.Y705 **is phosphorylated then** OSMR **unbinds** STAT3 **on** OSMR	19	
44	**if** STAT3.Y705 **is phospho and** STAT3 **is not dimer and** STAT3.gp130 **is not bound and** STAT3.LIFR **is not bound and** STAT3.OSMR **is not bound then** STAT3 **homodimerizes**	20	
	STAT3 shuttling		
45	**if** STAT3 **is in** 3 **and** STAT3 **is dimer and** STAT3.gp130 **is not bound and** STAT3.LIFR **is not bound and** STAT3.OSMR **is not bound then** STAT3 **relocates to** 4	21	
46	**if** STAT3 **is in** 4 **and** STAT3 **is dimer and** STAT3.PIAS3 **is not bound then** STAT3 **dephospho on** Y705	22	
47	**if** STAT3 **is in** 4 **and** STAT3 **is dimer and** STAT3.Y705 **is not phospho then** STAT3 **dehomodimerizes**	23	
48	**if** STAT3 **is in** 4 **and** STAT3 **is not dimer and** STAT3.Y705 **is not phospho then** STAT3 **relocates to** 3	24	
