# Supplementary material for: Narrative-based computational modelling of the Gp130/JAK/STAT signalling pathway
Source: BMC Syst Biol. 2009 Apr 15;3:40. doi: 10.1186/1752-0509-3-40 (PMC2678071; doi:10.1186/1752-0509-3-40)
Supplement: Additional file 8 — Table 8. Gp130/JAK/STAT pathway model: list of events (SOCS3 and PIAS3 inhibition). [file 1752-0509-3-40-S8.pdf]

id	description	react	alt
	SOCS3 synthesis, degradation and inhibition		
49	**if** STAT3 **is in** 4 **and** STAT3 **is dimer and** STAT3.Y705 **is phospho and** STAT3.PIAS3 **is not bound then** STAT3 **synthesises** SOCS3	25	
50	SOCS3 **degrades**	30	
51	**if** gp130.Y767 **is phospho and** gp130.SOCS3 **is not bound and** SOCS3 **is not bound and** gp130.STAT3 **is not bound then** SOCS3 **binds** gp130 **on** SOCS3	26	
52	**if** LIFR.Y981 **is phospho and** LIFR.SOCS3 **is not bound and** SOCS3 **is not bound and** LIFR.STAT3 **is not bound then** SOCS3 **binds** LIFR **on** SOCS3	26	
53	**if** OSMR.Y917 **is phospho and** OSMR.SOCS3 **is not bound and** SOCS3 **is not bound and** OSMR.STAT3 **is not bound then** SOCS3 **binds** OSMR **on** SOCS3	26	
54	**if** gp130.Y767 **is phospho and** gp130.SOCS3 **is bound and** SOCS3 **is bound and** gp130.STAT3 **is not bound then** SOCS3 **unbinds** gp130 **on** SOCS3	27	
55	**if** LIFR.Y981 **is phospho and** LIFR.SOCS3 **is bound and** SOCS3 **is bound and** LIFR.STAT3 **is not bound then** SOCS3 **unbinds** LIFR **on** SOCS3	27	
56	**if** OSMR.Y917 **is phospho and** OSMR.SOCS3 **is bound and** SOCS3 **is bound and** OSMR.STAT3 **is not bound then** SOCS3 **unbinds** OSMR **on** SOCS3	27	
	PIAS3 inhibition		
57	**if** STAT3 **is in** 4 **and** STAT3 **is dimer and** STAT3.Y705 **is phospho and** STAT3.PIAS3 **is not bound and** PIAS3 **is not bound then** PIAS3 **binds** STAT3 **on** PIAS3	28	
58	**if** STAT3 **is in** 4 **and** STAT3 **is dimer and** STAT3.Y705 **is phospho and** STAT3.PIAS3 **is bound and** PIAS3 **is bound then** PIAS3 **unbinds** STAT3 **on** PIAS3	29	
