# Supplementary figures and images for: Narrative-based computational modelling of the Gp130/JAK/STAT signalling pathway
Source: BMC Syst Biol. 2009 Apr 15;3:40. doi: 10.1186/1752-0509-3-40 (PMC2678071; doi:10.1186/1752-0509-3-40)

**A**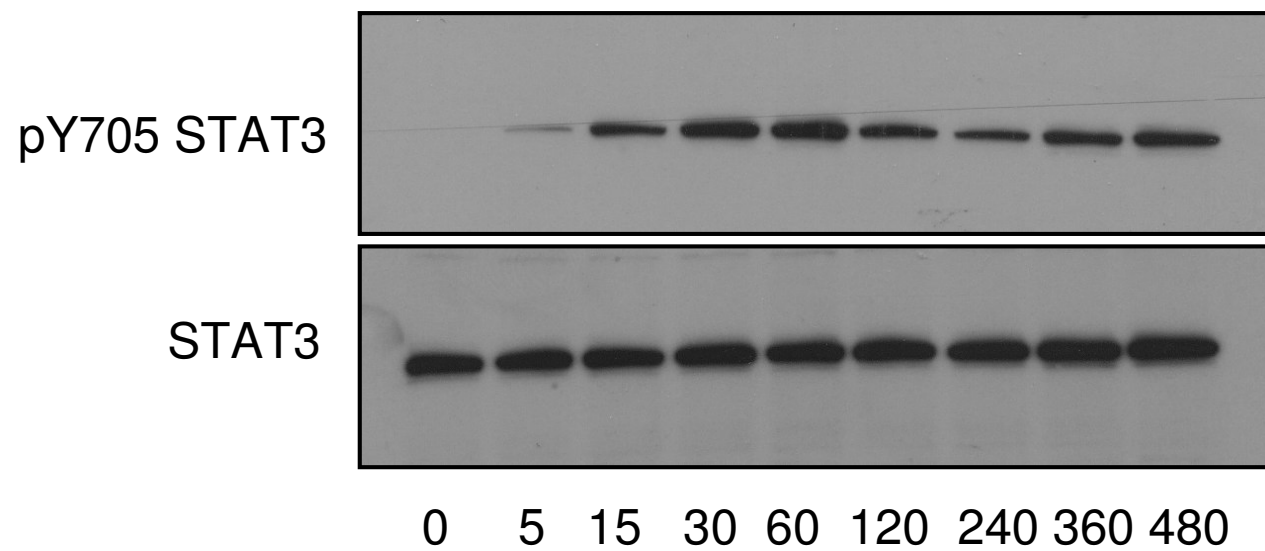**B**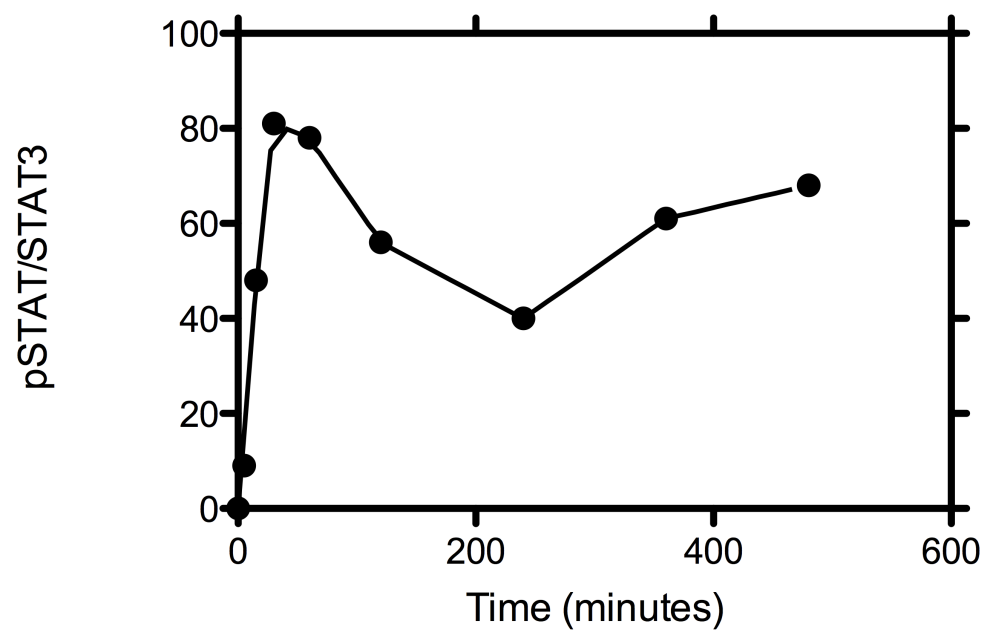**C**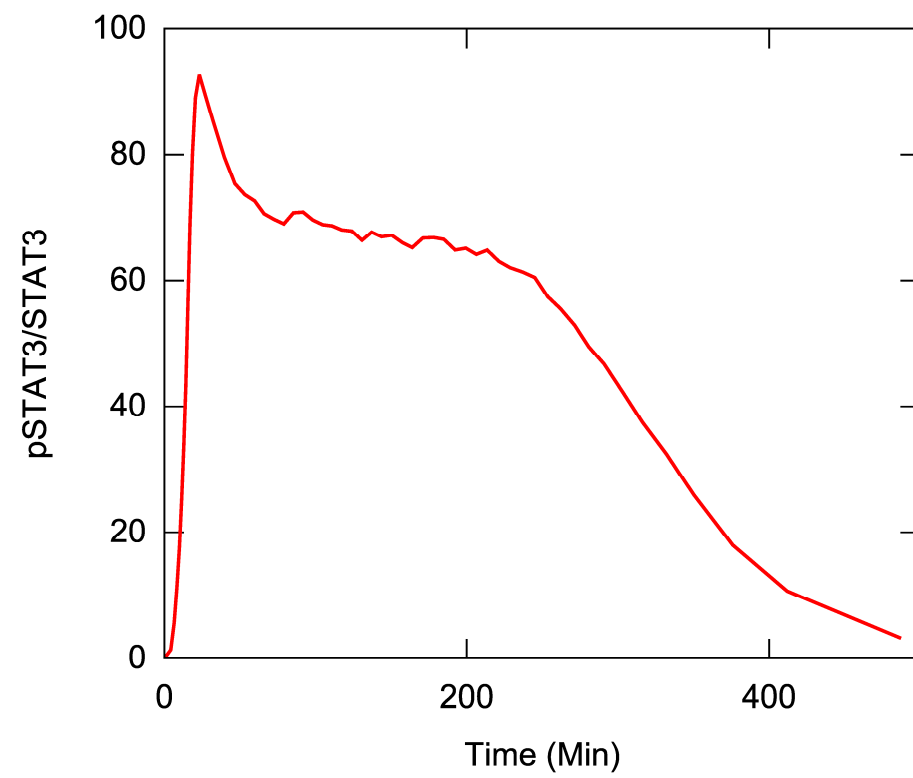

Supplement: Additional file 9 — Time-evolution of STAT3 phosphorylation. (A) Levels of phospho-STAT3 (upper panel) and total STAT3 (lower panel) after stimulation with oncostatin M. (B) Densitometric analysis of immunoblots showing the time-course of phospho-STAT3/STAT3 ratios. (C) Simulation time-course of phospho-STAT3/STAT3 ratios. [file 1752-0509-3-40-S9.pdf]

**A**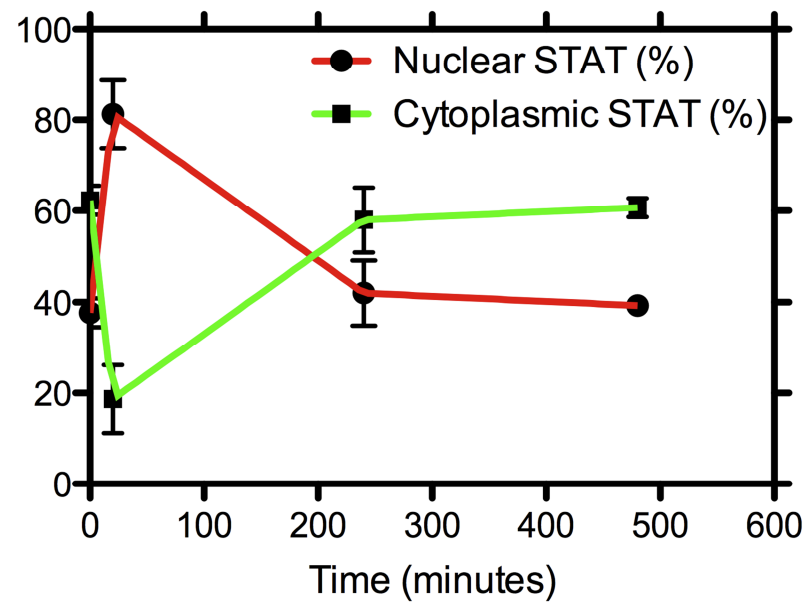**C**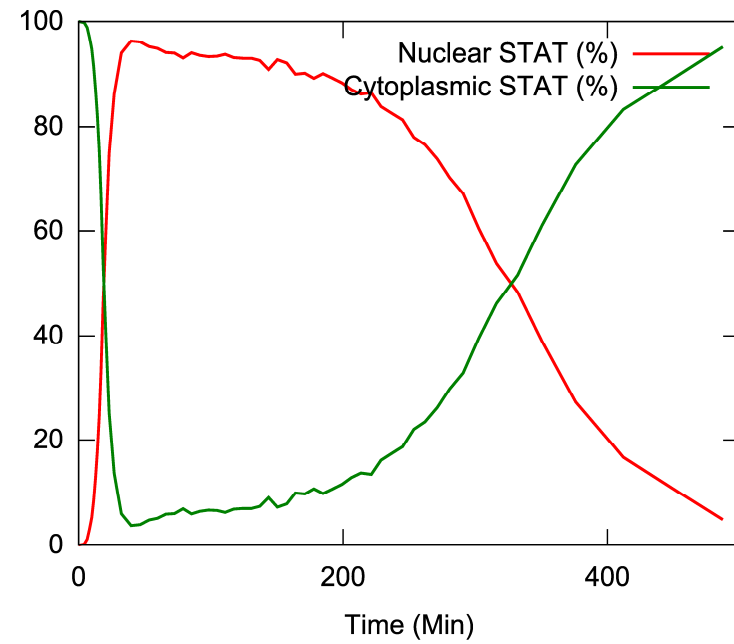**B**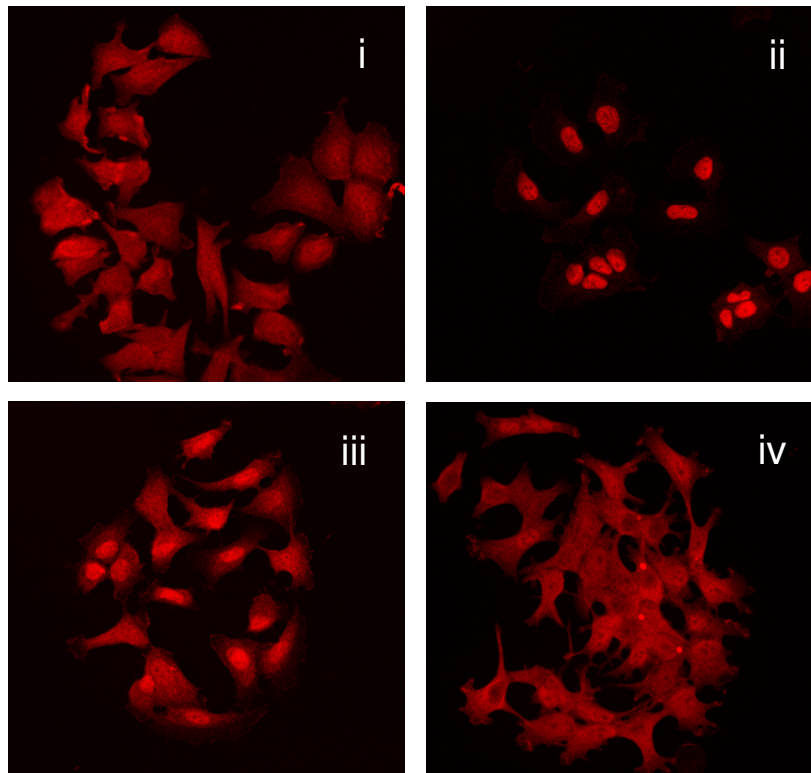

Supplement: Additional file 10 — Nuclear localisation of STAT3. (A) Time-course of STAT3 nuclear/cytoplasmic localisation. (B) Images representative of STAT3 localisation after stimulation with Oncostatin M: (i) control; (ii) 20 minutes; (iii) 4 hours; (iv) 8 hours. (C) Simulation time-course of STAT3 nuclear/cytoplasmic localisation. [file 1752-0509-3-40-S10.pdf]
